# Sodium Alginate/UiO-66-NH_2_ Nanocomposite for Phosphate Removal

**DOI:** 10.3390/nano14141176

**Published:** 2024-07-10

**Authors:** Xiaohang Lin, Yuzhu Xiong, Fuping Dong

**Affiliations:** Department of Polymer Materials and Engineering, College of Materials and Metallurgy, Guizhou University, Guiyang 550025, China; 18185070974@163.com (X.L.); yzxiong@gzu.edu.cn (Y.X.)

**Keywords:** sodium alginate, UiO-66-NH_2_, phosphate removal, porous nanocomposite

## Abstract

Environmental pollution of phosphorus is becoming increasingly concerning, and phosphate removal from water has become an important issue for controlling eutrophication. Modified metal–organic framework (MOF) materials, such as UiO-66-NH_2_, are promising adsorbents for phosphate removal in aquatic environments due to their high specific surface area, high porosity, and open active metal sites. In this study, a millimeter-sized alginate/UiO-66-NH_2_ composite hydrogel modified by polyethyleneimine (UiO-66-NH_2_/SA@PEI) was prepared. The entrapping of UiO-66-NH_2_ in the alginate microspheres and its modification with PEI facilitate easy separation in addition to enhanced adsorption properties. The materials were characterized by SEM, FTIR, XRD, and BET. Static, dynamic, and cyclic adsorption experiments were conducted under different pH, temperature, adsorbent dosage, and initial concentration conditions to assess the phosphate adsorption ability of UiO-66-NH_2_/SA@PEI. Under optimal conditions of 65 °C and pH = 2, 0.05 g UiO-66-NH_2_/SA@PEI adsorbed 68.75 mg/g, and the adsorption rate remained at 99% after five cycles of UiO-66-NH_2_/SA@PEI. These results suggest that UiO-66-NH_2_/SA@PEI composite materials can be used as an effective adsorbent for phosphate removal from wastewater.

## 1. Introduction

Phosphorus pollution resulting from the extensive production and mismanagement of pesticides, detergents, and phosphate fertilizers is becoming more and more serious [1]. Phosphorus manifests in water bodies in multiple forms, such as phosphates, polyphosphates, and organic phosphorus. Excessive phosphate in water can cause eutrophication, leading to the growth of harmful blue-green algae, water quality decline, abnormal death of fish/invertebrates, and ultimately disturbing the local ecological balance, causing harm to the ecological environment [2]. The removal of phosphate from water bodies is essential to enhance the ecological environment [3].

Various methods, such as precipitation, adsorption, ion exchange, and biological processes have been utilized to remediate phosphate pollution [4,5,6,7,8]. Phosphorus, a non-renewable resource, is significant not only for preventing phosphorus pollution but also for recycling phosphorus resources from wastewater [9]. The adsorption method facilitates the dual purpose of removing contaminants from water bodies and converting them into fertilizer for agricultural use. The development of efficient adsorbents and techniques for phosphate removal holds significant practical importance for the protection of water resources and the environment [10].

In the adsorption of phosphate in wastewater, the oxides and hydroxides of trivalent and tetravalent metals like Fe, Al, Mn, La, Ce, and Zr demonstrate rapid kinetics and superior adsorption capacity [11]. Metal–organic frameworks (MOFs) have recently attracted significant attention for their potential in phosphate removal applications. With high surface areas, controllable pore dimensions, substantial porosity, unique architecture, diverse functional groups, and stability under varying temperature and pH conditions, MOFs are well suited as adsorbents for phosphate recovery compared to conventional oxides and hydroxides [12]. In fact, MOFs have exhibited enhanced phosphate adsorption capabilities when constructed using appropriate metal and organic ligands, which are attributed to their superior specific surface areas and stability [13,14,15,16,17]. Shams et al. developed a novel hybrid adsorbent, cubic zeolitic imidazolate framework-8 (ZIF-8), and assessed its efficacy in removing phosphate from aqueous solutions, revealing a notable adsorption capacity of 38.22 mg/g [18]. Zirconium-based MOFs, compared to other metals, exhibit ordered porous structures, diverse functional groups, excellent ion exchange/adsorption capabilities, and outstanding water stability [19,20,21,22,23]. Consequently, they are being investigated for phosphate adsorption and removal in water.

However, the particle agglomeration of MOFs often causes a lack of full contact between the active adsorption sites and the adsorbate, ultimately limiting the adsorption efficiency of the materials [24]. MOFs typically exist in powder form, posing challenges for their recyclability after being utilized for phosphate adsorption [25,26]. Sodium alginate (SA), a natural and cheap polysaccharide polymer, has a strong ability to bond with multivalent metal ions in aqueous solutions, and it forms a stable hydrogel by cross-linking [27,28,29,30]. In this study, UiO-66-NH_2_ nanoparticles were compounded with sodium alginate, gelled with Ca^2+^, surface grafted with polyethyleneimine, and subsequently exchanged with Zr^4+^ to produce UiO-66-NH_2_/SA@PEI composites. The impact of pH, temperature, adsorbent dosage, and initial concentration on phosphate removal using the composite material was systematically investigated through batch experiments, with a discussion on its static/dynamic adsorption and regeneration performance.

## 2. Materials and Methods

### 2.1. Chemicals

Sodium alginate, zirconium oxychloride octahydrate (99%), polyethylene imine (PEI, M_W_ = 10,000, 99%), glacial acetic acid (99.5%), N, N-dimethylformamide (99.5%), zirconium chloride (98%), ammonium molybdate (99.8%), potassium antimony tartrate hemihydrate (98%), and potassium dihydrogen phosphate (99.5%) were purchased from Aladdin, Shanghai, China; anhydrous calcium chloride was obtained from Xilong Chemical, Shantou, China; 2-Amino para benzyl dimethyl (98%) and L-ascorbic acid (>99.0%) were acquired from Macklin, Shanghai, China; sulfuric acid and hydrochloric acid were purchased from Chuandong Chemical, Chongqing, China; and sodium hydroxide (AR) was obtained from Chengdu Jinshan Chemical Reagent Company (Chengdu, China). Deionized water (DW) was produced by an ultra-pure water instrument.

### 2.2. Synthesis of UiO-66-NH_2_/SA@PEI

UiO-66-NH_2_ nanoparticles were prepared according to the previous literature [31]. To synthesize UiO-66-NH_2_/SA, 1.5 g of UiO-66-NH_2_ was dispersed in 50 mL of water, followed by the addition of 1 g of sodium alginate after ultrasonication. The resulting mixture was heated at 100 °C in a water bath and stirred for 12 h to form a viscous suspension. This suspension was then slowly added dropwise into a 5% (*w/v*) CaCl_2_ solution and allowed to react for 12 h with gentle stirring to form hydrogel beads. Subsequently, the beads underwent three water washes to remove residual CaCl_2_, followed by immersion in a 2% polyethyleneimine 10000 (PEI-10000) solution at 50 °C for 1 h, and then rinsed with water. Finally, the hydrogel beads were immersed in a 50 mL ZrOCl_2_ aqueous solution (5% wt) for 12 h to displace the calcium ions within the beads, followed by three water washes and freeze-drying for preservation.

### 2.3. Adsorption of Phosphate by UiO-66-NH_2_/SA@PEI

The content of phosphate in the solution was determined by molybdenum antimony anti-spectrophotometry. An appropriate amount of UiO-66-NH_2_/SA@PEI was added to 25 mL of a phosphate solution whose pH had been adjusted with NaOH and HCl. Following this, an adsorption experiment was conducted at a predetermined temperature. Subsequently, the adsorption capacity of UiO-66-NH_2_/SA@PEI could be calculated with Formulas (1) and (2).

(1) $$Q_{e}=\frac{\left(C_{0}-C_{e}\right)\cdot V}{M}$$

(2) $$Removal\;rate\;\%=\frac{C_{0}-C_{t}}{C_{0}}\times 100\%$$

*Q_e_* is the equilibrium adsorption capacity of UiO-66-NH_2_/SA@PEI, mg/g; *C*_0_ is the initial concentration of phosphate solution, mg/L; *C_e_* is the concentration of phosphate solution at equilibrium, mg/L; *V* is the solution volume, mL; and *M* is the mass of the adsorbent, g.

For recycling adsorption, UiO-66-NH_2_/SA@PEI was immersed in a 0.1 M NaOH solution for 30 min and then washed with water for further adsorption experiments.

A dynamic adsorption system of UiO-66-NH_2_/SA@PEI was designed with a peristaltic pump and glass beads, allowing the liquid to enter and exit the column from the bottom. The adsorption column had an inner diameter of 1 cm and a length of 20 cm, with the adsorbent positioned in the middle and glass beads filling the top and bottom. The adsorption efficiency of the UiO-66-NH_2_/SA@PEI sample was studied under different concentrations of phosphate solutions and peristaltic pump flow rates.

### 2.4. Characterizations

The morphology of the adsorbent was observed by scanning electron microscopy (SEM) using an FEI-SEM system (FEI Helios Nanolab 600i, Hillsboro, OR, USA) operating at 15 kV. Before measurement, all samples were sprayed with a thin gold film. Fourier transform infrared spectroscopy (FTIR) was recorded on a Perkin–Elmer Spectrum GX-spectrophotometer (Waltham, MA, USA) with a spectral resolution of 1 cm^−1^ and a scan number of 32. X-ray diffraction (XRD) patterns were recorded using a Philips diffractometer with a Geiger counter (Eindhoven, The Netherlands). The X-ray tube was operated at 40 kV and 30 mA (Cu Kα radiation with Ni filter, λ = 1.5406 Å) with a scan speed of 1°/min. Nitrogen adsorption–desorption measurements (ASAP 2046, Micromeritics, Norcross, GA, USA) were performed at 77 K to assess their Brunauer–Emmett Teller (BET) surface areas. UV–visible spectra were recorded using a UV-2700 spectrophotometer (Shimadzu, Kyoto, Japan) with 1 cm quartz cuvettes.

## 3. Results

### 3.1. Characterization

**Figure 1:**
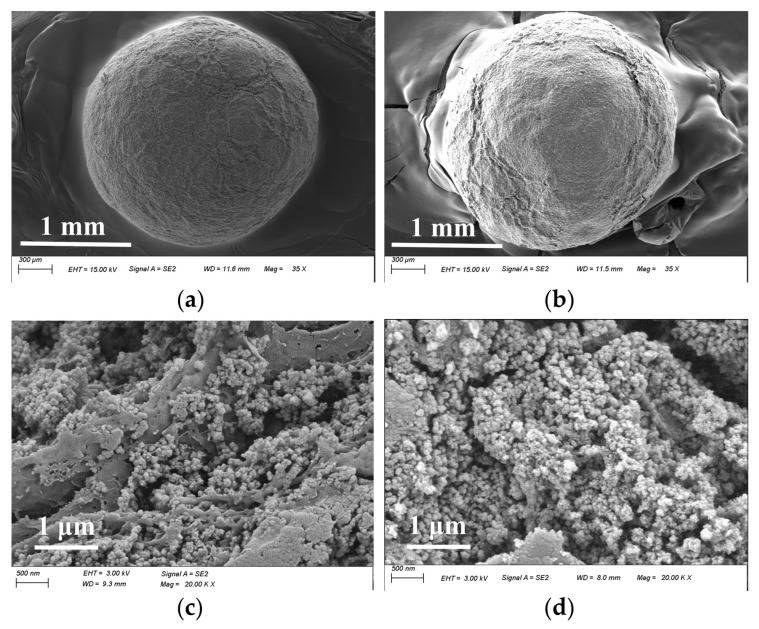
The SEM images of UiO-66-NH_2_/SA@PEI before (**a**,**c**) and after the phosphate adsorption (**b**,**d**).

The morphology of the material was analyzed using a scanning electron microscope before and after the phosphate adsorption. The results revealed no significant differences in size or morphology and only a slight increase in surface roughness (Figure 1a,b). Examination of the cross-section images showed that the structure of the adsorbent remained intact after the phosphate adsorption (Figure 1c,d). Furthermore, a close connection between the UiO-66-NH_2_ nanoparticles and the SA network structure was observed.

**Figure 2:**
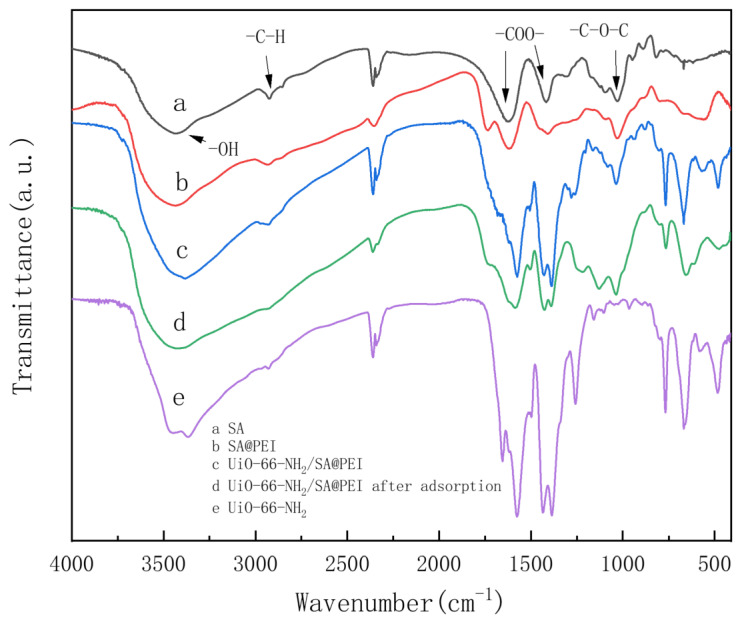
FTIR spectra of (**a**) SA, (**b**) SA@PEI, (**c**,**d**) UiO-66-NH_2_/SA@PEI before and after adsorption, (**e**) UiO-66-NH_2_.

Figure 2 shows the FT-IR spectra of the SA, UiO-66-NH_2_, SA@PEI and UiO-66-NH_2_/SA@PE materials before and after the adsorption. It can be observed that the stretching vibration peak of -OH in SA appeared at 3430 cm^−1^, while the stretching vibration of -CH_2_ occurred at 2930 cm^−1^. Furthermore, the absorption peaks at 1638 cm^−1^ and 1410 cm^−1^ were attributed to the stretching vibration of -COOH [32]. Additionally, the absorption peak at 1031 cm^−1^ was caused by the stretching vibration of -COC. After the PEI was grafted, the peak position at 3000–3500 cm^−1^ shifted to the right, and the peak value was enhanced, indicating an increase in the NH_2_ groups [33]. The FTIR spectrum of UiO-66-NH_2_/SA@PEI displayed the characteristic peaks of UiO-66-NH_2_ at various positions, which suggests that UiO-66-NH_2_/SA was successfully compounded. A comparison between Figure 2c and Figure 2d reveals a significant enhancement of the peak at 1047 cm^−1^, with the absorption peak region for the P-O stretching vibrations located between 1000 and 1100 cm^−1^, indicating successful phosphate adsorption [34].

**Figure 3:**
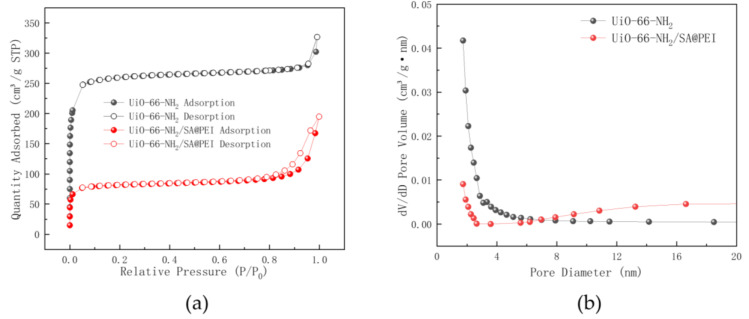
(**a**) N_2_ adsorption–desorption isotherms and (**b**) pore size distribution curves for UiO-66-NH_2_ and UiO-66-NH_2_/SA@PEI.

The adsorption–desorption isotherms and pore size distribution curves of UiO-66-NH_2_ and UiO-66-NH_2_/SA@PEI are shown in Figure 3a,b. The N_2_ adsorption–desorption isotherm of UiO-66-NH_2_ exhibited a Type II curve. Upon composite formation with SA@PEI, UiO-66-NH_2_/SA@PEI displayed a distinct hysteresis loop in the P/P_0_ range of 0.6–1.0. The specific surface area decreased from 1062.2 m^2^/g to 325.1 m^2^/g, while the pore volume reduced from 0.5 cm^3^/g to 0.3 cm^3^/g. Furthermore, the average pore diameter increased from 1.9 nm to 3.7 nm, facilitating enhanced diffusion of phosphates within the material and providing additional adsorption sites for potential applications.

**Figure 4:**
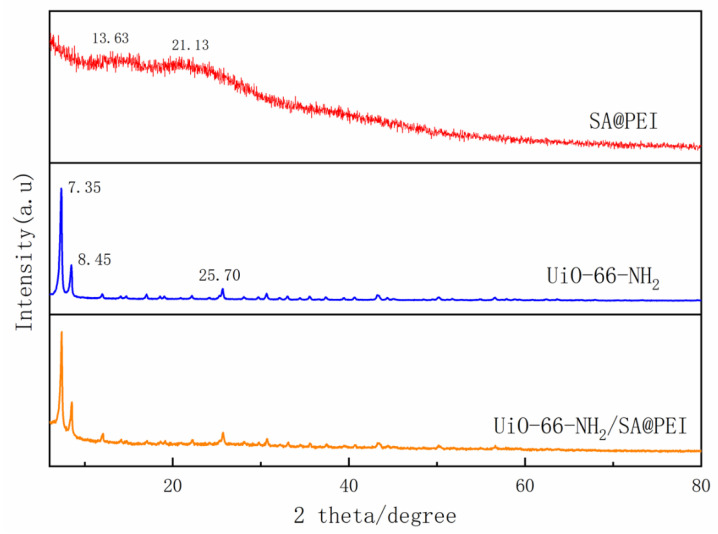
XRD patterns of SA@PEI, UiO-66-NH_2_ and UiO-66-NH_2_/SA@PEI.

Figure 4 illustrates that UiO-66-NH_2_ exhibited strong characteristic peaks at 7.35°, 8.45°, and 25.7°, while SA@PEI had weaker peaks at 13.63° and 21.31° [35]. The spectrum for UiO-66-NH_2_/SA@PEI revealed that the characteristic peaks of UiO-66-NH_2_ remained intact, indicating that the combination of sodium alginate did not change the crystal structure of UiO-66-NH_2_.

### 3.2. The Adsorption Capacity of UiO-66-NH_2_/SA@PEI under Different Conditions

**Figure 5:**
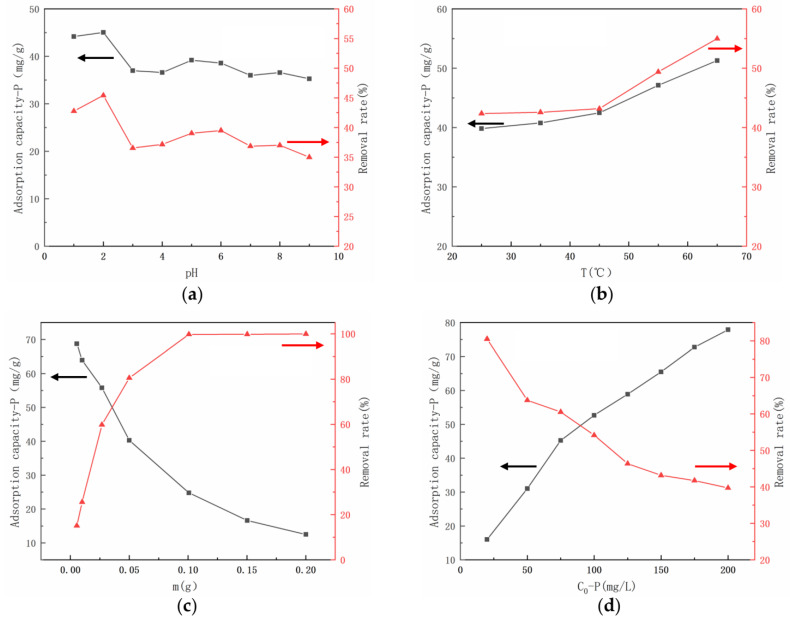
The adsorption capacity and removal rate of the phosphate adsorption by UiO-66-NH_2_/SA@PEI under different conditions: (**a**) pH, (**b**) temperature, (**c**) dosage of adsorbent (**d**) initial phosphate concentration.

In order to determine the phosphate adsorption capacity of UiO-66-NH_2_/SA@PEI from water, experiments were conducted under various conditions, including pH, temperature, adsorbent quantity, and initial phosphate concentration. To investigate the impact of pH on adsorption capacity, the pH was varied from 1 to 9 using 0.05 g of adsorbent, an initial phosphate concentration of 100 mg/L, a volume of 12.5 mL, and an adsorption temperature of 25 °C. Figure 5a illustrates the consistently high adsorption capacity of UiO-66-NH_2_/SA@PEI for phosphate across the pH range of 1–9. The positively charged surface of UiO-66-NH_2_ at lower pH levels enhanced its affinity for negatively charged H_2_PO_4_^−^/HPO_4_^2−^, while the protonation of -NH_2_ to -NH_3_^+^ further strengthened the electrostatic adsorption of phosphate ions.

Adsorption experiments were conducted at temperatures of 25 °C, 35 °C, 45 °C, 55 °C, and 65 °C using a dosage of 0.05 g of UiO-66-NH_2_/SA@PEI, a phosphate concentration of 100 mg/L, a pH of 2, and a volume of 12.5 mL. Figure 5b depicts the relationship between the adsorption capacity, removal rate, and temperature of phosphate adsorbed by UiO-66-NH_2_/SA@PEI. The results indicated a direct correlation between temperature and the adsorption capacity of phosphate by the adsorbent, increasing from 39.8 mg/g to 51.3 mg/g. Additionally, the removal rate rose from 42.4% to 55.1%. These findings suggest that the adsorption of phosphate by UiO-66-NH_2_/SA@PEI is an endothermic process, as higher temperatures enhanced the adsorption capacity.

The effect of varying amounts of adsorbent on phosphate removal was examined using an initial phosphate solution of 100 mg/L, a 25 mL volume, a pH of 2, and a temperature of 65 °C. Different dosages of adsorbent—0.0055 g, 0.01 g, 0.0265 g, 0.05 g, 0.1 g, 0.15 g, and 0.2 g—were employed in the investigation. As shown in Figure 5c, upon increasing the dosage of UiO-66-NH_2_/SA@PEI, the adsorption capacity of the adsorbent reached 68.75 mg/g at a dosage of 0.0055 g. Increasing the dosage of the adsorbent led to an increase in adsorption sites; however, the phosphate content in the system remained constant, resulting in internal competition within the adsorbent. This competition led to a decrease in the amount of phosphate adsorbed per unit of adsorbent, hence reducing the adsorption capacity. The removal efficiency of UiO-66-NH_2_/SA@PEI for phosphorus initially increased rapidly, then plateaued. At a dosage of 0.1 g, the removal efficiency of UiO-66-NH_2_/SA@PEI in the adsorption system reached 99.76%. Beyond a dosage of 0.1 g, the removal efficiency remained relatively constant, indicating an excess of adsorption sites in the adsorbent compared to the phosphate content in the system.

An investigation into the effect of initial concentration on the removal of P using UiO-66-NH_2_/SA@PEI (a dosage of 0.05 g, a pH of 2, a volume of 12.5 mL, and an adsorption temperature of 65 °C) revealed that the adsorption amount increased with an increase in the initial concentration (Figure 5d). However, the rate of increase in the adsorption amount decreased with a higher initial concentration, resulting in a decrease in the removal rate of the adsorbent [36]. Higher initial concentrations led to an increase in adsorption capacity, with the rate of increase gradually decreasing. However, higher initial concentrations also reduced the removal efficiency of the adsorbent. The high initial phosphate concentrations facilitated the binding of phosphate ions to the adsorption sites on UiO-66-NH_2_/SA@PEI. As the number of adsorption sites was limited once saturation was reached, no further adsorption occurred. Consequently, higher initial concentrations resulted in lower phosphate removal rates.

### 3.3. Adsorption Kinetics of Phosphate by UiO-66-NH_2_/SA@PEI

To investigate the adsorption kinetics of phosphate on UiO-66-NH_2_/SA@PEI, kinetic data were fitted using pseudo-first-order (3), pseudo-second-order (4), and Elovich (5) models with varying initial phosphate concentrations.

(3) $$Q_{t}=Q_{e}\left(1-e^{-k_{1}t}\right)$$

(4) $$Q_{t}=k_{2}Q_{e}^{2}t/\left(1+k_{2}Q_{e}t\right)$$

(5) $$Q_{t}=[\ln(\alpha \beta )+\ln t]/\beta$$

*Q_t_* is the adsorption amount of UiO-66-NH_2_/SA@PEI at time *t*, mg-P/g; *Q_e_* is the adsorption amount at adsorption equilibrium, mg-P/g; *k* is the adsorption constant of each model, *k*_1_-h^−1^, *k*_2_-g/(mg·h); *α* is the adsorption rate constant, mg/(mg·h); and *β* is the adsorbent surface coverage and chemical adsorption activation of energy-related parameters, g/mg.

**Figure 6:**
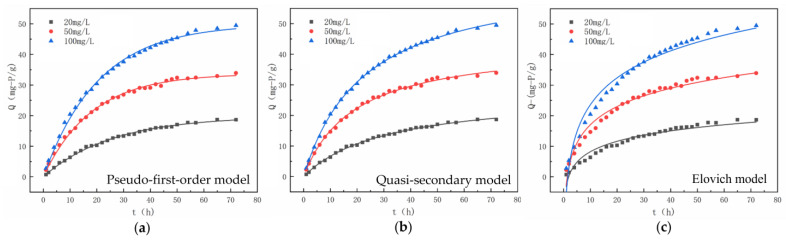
Model fitting curves for phosphate adsorption by UiO-66-NH_2_/SA@PEI, (**a**) Pseudo-first-order model, (**b**) Quasi-secondary model and (**c**) Elovich model.

The curve fitting for the adsorption kinetic data of UiO-66-NH_2_/SA@PEI on phosphate is illustrated in Figure 6, with corresponding fitting parameters detailed in Table 1. The correlation coefficients of the pseudo-first-order and pseudo-second-order kinetic models at initial concentrations of 20 mg/L, 50 mg/L, and 100 mg/L exceeded 0.99, indicating a strong correlation. Notably, the pseudo-second-order kinetic model showed a higher coefficient of determination compared to the pseudo-first-order kinetic model, suggesting that the adsorption process was primarily governed by chemical adsorption. Phosphate was effectively adsorbed onto the materials through a chemical reaction, also as evidenced by the emergence of a peak at 1047 cm^−1^, corresponding to the P-O stretching vibration peak following phosphate adsorption in the FTIR spectrum (Figure 2d).

### 3.4. The Adsorption Isotherm of Phosphate by UiO-66-NH_2_/SA@PEI

**Figure 7:**
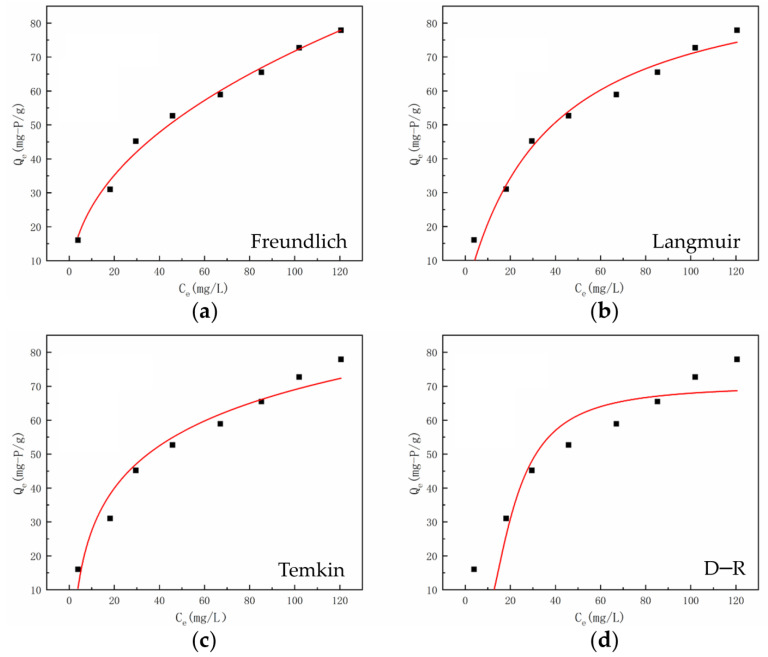
The adsorption isotherm of phosphate by UiO-66-NH_2_/SA@PEI, (**a**) Freundlich model, (**b**) Langmuir model, (**c**) Temkin model and (**d**) D–R model.

The adsorption isotherm, illustrating the equilibrium concentration (*C_e_*) and adsorbed amount (*Q_e_*) at a given temperature and pH, was analyzed using the Langmuir (6), Freundlich (7), Temkin (8), and Dubinin–Radushkevich (DR) (9) models. The formulas, associated parameters, and fitting curves for these models are presented in Table 2 and Figure 7.

(6) $$Q_{e}=Q_{m}K_{L}C_{e}/\left(1+K_{L}C_{e}\right)$$

(7) $$Q_{e}=K_{F}C_{e}^{1/n_{F}}\;$$

(8) $$Q_{e}=B\ln K_{T}+B\ln C_{e}$$

(9) $$Q_{e}=Q_{m}exp\left\{-K_{D}{\left[RTln\left(1+\frac{1}{C_{e}}\right)\right]}^{2}\right\}$$

*Q_e_* is the adsorption amount at adsorption equilibrium, mg-P/g; *K* is the adsorption constant of each model, *K_L_*-L/mg, *K_F_*-mg/g, *K_T_*-L/mg, and *K_D_*-mol^2^/kJ^2^; *Q_m_* is the theoretical maximum adsorption capacity, mg-P/g; *n_F_* is the Freundlich model constant, which is related to the adsorption strength; *B* is the Temkin model constant related to the heat of adsorption, kJ/mol; *C_e_* is the concentration at equilibrium, mg/L; *R* is the ideal gas constant, 8.314 kJ/mol/K; and *T* is the absolute temperature, K.

As shown in Table 2 and Figure 7, the adsorption isotherms of UiO-66-NH_2_/SA@PEI were analyzed using the Langmuir, Freundlich, Temkin, and Dubinin–Radushkevich models. The correlation coefficients (R^2^) for the Langmuir, Freundlich, and Temkin models were all above 0.9, indicating strong correlations [37]. However, the Dubinin–Radushkevich model had an R^2^ of only 0.8157, making it unsuitable for data fitting. The Langmuir model predicted a maximum theoretical adsorption capacity (*Q_m_*) of 96.8 mg/g for P on UiO-66-NH_2_/SA@PEI. The Freundlich model exhibited the highest correlation coefficient (R^2^ = 0.9901) compared to the Langmuir (R^2^ = 0.9680) and Temkin (R^2^ = 0.94701) models, suggesting non-uniform surface adsorption. The adsorption data demonstrated a good fit with the Freundlich model, showing a 1/*n_F_* value of 0.4431, which is below 0.5. This value indicates the presence of chemical adsorption between phosphate and the adsorbent, highlighting the ease of phosphate adsorption. The Temkin model showed a strong correlation, implying a significant role of electrostatic forces in the adsorption process.

### 3.5. Cyclic Adsorption Performance of UiO-66-NH_2_/SA@PEI

**Figure 8:**
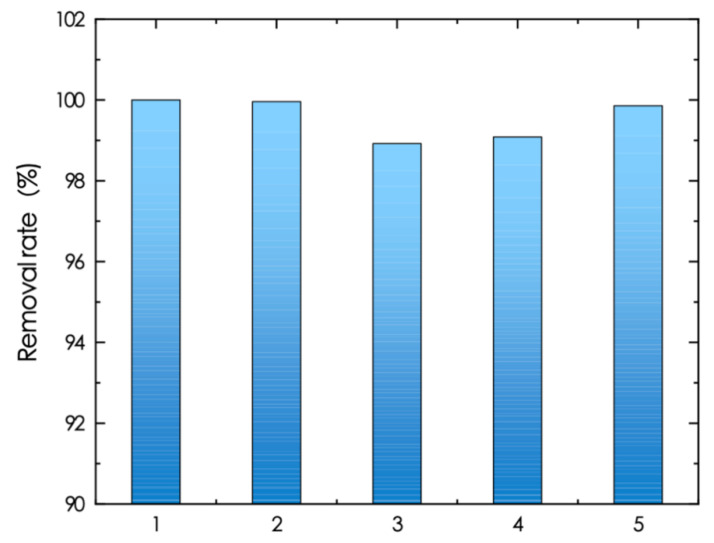
Cyclic adsorption performance of UiO-66-NH_2_/SA@PEI.

An experiment was conducted to assess the cyclic adsorption capacity of UiO-66-NH_2_/SA@PEI following regeneration, utilizing NaOH for desorption. Re-adsorption took place under consistent initial phosphate concentration, adsorption temperature, and pH conditions. After five cycles, the removal rate still reached 99%, indicating that UiO-66-NH_2_/SA@PEI has a good cyclic adsorption capacity as a phosphate adsorbent (Figure 8).

### 3.6. Dynamic Adsorption Capacity

**Figure 9:**
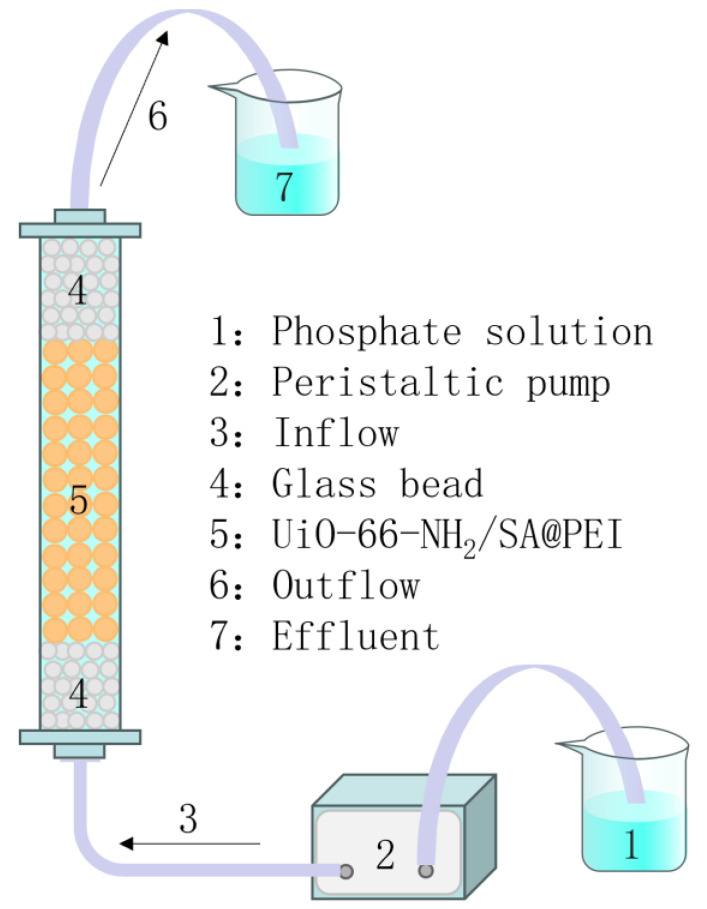
Schematic diagram of fixed-bed column used in dynamic adsorption study of phosphate onto UiO-66-NH_2_/SA@PEI.

To further examine the dynamic adsorption capacity of UiO-66-NH_2_/SA@PEI, experiments were conducted at different initial concentrations and flow rates. As depicted in Figure 9, a phosphate solution was delivered into a fixed-bed column containing UiO-66-NH_2_/SA@PEI, using a peristaltic pump for dynamic adsorption under various conditions.

The Thomas model (10) was used to fit the results of the experiments. The removal rate of phosphate was calculated by Formula (11).

(10) $$\frac{C_{t}}{C_{0}}=\frac{1}{1+e^{\frac{K_{T}Qm}{v}-K_{T}C_{0}t}}$$

(11) $$R\;\%=\frac{C_{0}vt_{e}/1000}{\frac{v}{1000}\int_{0}^{t_{e}}\left(C_{0}-C_{t}\right)dt}\times 100\%$$

*C*_0_ is the initial concentration of the inlet water, mg-P/L; *C_t_* is the outlet solution concentration at time *t*, mg-P/L; *K_T_* is the Thomas model constant, mL/(min·mg); *v* is the flow rate of the solution, mL/min; *Q* is the adsorption capacity of the adsorbent, mg-P/g; *m* is the mass of the adsorbent, g; R (%) is the removal rate; and the depletion point *t_e_* (min) of the adsorption column is the time point when *C_t_*/*C*_0_ = 95%.

**Figure 10:**
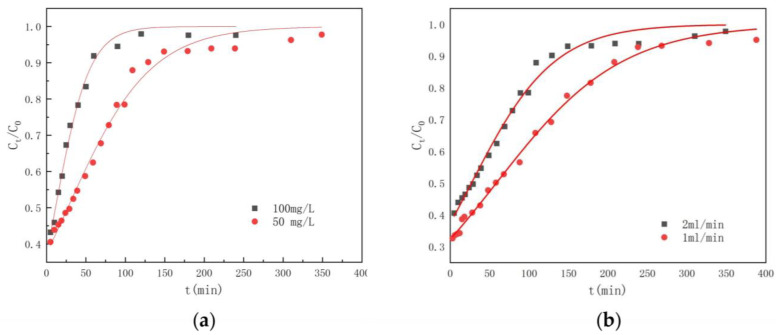
Thomas model fitting for dynamic adsorption capacity of UiO-66-NH_2_/SA@PEI under different conditions, (**a**) initial phosphate concentration and (**b**) water inlet rate.

The Thomas model was used to fit the adsorption data of UiO-66-NH_2_/SA@PEI with a dosage of 2 g, initial concentrations of 50 mg/L and 100 mg/L and water inlet rates of 1 mL/min and 2 mL/min, respectively. The results showed that the correlation coefficient R^2^ fitted by the model was greater than 0.95, indicating a strong correlation (Table 3, Figure 10). According to the Thomas model, it can be seen that as the flow rate increased, the adsorption capacity decreased and the rate constant (*K_T_*) increased, suggesting that a lower flow rate is more conducive to adsorption. This is because a lower flow rate will increase the contact time between the adsorbent and the solution, thus improving the adsorption effect and increasing the rate constant. Additionally, as the initial concentration increased, the adsorption capacity decreased and the rate constant increased. This is because a higher concentration increases the concentration difference between the liquid and the adsorbent, thus increasing the driving force transmitted to it. At the same time, the high concentration occupies the adsorption sites faster, resulting in a decrease in the rate constant and a decrease in the *t_e_*.

### 3.7. Phosphate Adsorption Mechanism

The adsorption mechanism of UiO-66-NH_2_/SA@PEI involves numerous metal sites, with Zr present in the form of Zr_6_O_4_(OH)_4_ carrying a positive charge. This allows for electrostatic interactions with negatively charged phosphate ions. At low pH, the -OH groups on Zr_6_O_4_(OH)_4_ are more reactive, enabling phosphate ions to adsorb by displacing -OH groups on Zr [38]. Additionally, UiO-66-NH_2_/SA@PEI provides another adsorption site through positively charged amine groups on UiO-66-NH_2_ and PEI, which interact electrostatically with phosphate ions for adsorption [12]. In UiO-66-NH_2_/SA@PEI material, the predominant adsorption sites exposed to the solvent are zirconium metal and amino groups. The presence of water is essential for the adsorption process. When the adsorbent input is equal, environments rich in water facilitate full accessibility of the adsorption sites to the solvent, thereby enhancing the adsorption capacities and accelerating saturation attainment [39].

The composite porous structure of UiO-66-NH_2_/SA@PEI enhances phosphate mass transfer and diffusion within its pores, thereby increasing adsorption capacity on the UiO-66-NH_2_ surface. Despite not having the highest specific surface area, the UiO-66-NH_2_/SA@PEI prepared in this study exhibited superior adsorption capacity due to its porosity and improved particle dispersion, resulting in a greater number of available adsorption sites, as illustrated in Table 4.

## 4. Conclusions

A UiO-66-NH_2_/SA@PEI composite material was synthesized for phosphate adsorption. After the formation of the composite materials, the UiO-66-NH_2_ nanoparticles exhibited good dispersibility and a close association with the SA network. This composite material not only protected and stabilized UiO-66-NH_2_ but also resolved the issue of recyclability inherent in UiO-66-NH_2_ as an adsorbent. With a high concentration of Zr metal sites and amino active sites, the composite material demonstrated efficient adsorption of phosphate ions in aqueous solutions. Under optimal conditions of 65 °C and pH = 2, 0.05 g UiO-66-NH_2_/SA@PEI achieved an adsorption of 68.75 mg/g, and the adsorption rate remained at 99% after five cycles. The higher temperature favored adsorption, indicating an endothermic reaction. Data fitting revealed that the pseudo-second-order and Freundlich models best matched the experimental results. The cyclic adsorption tests demonstrated UiO-66-NH_2_/SA@PEI’s strong re-adsorption capability, highlighting its potential as a phosphate adsorbent.

## Figures and Tables

**Table 1:** Fitting parameters of the kinetic model of UiO-66-NH_2_/SA@PEI for phosphate adsorption.

Kinetic Model		Initial Concentration of Phosphate Solution (mg/L)		
		20	50	100
Pseudo-first-order model	*k*_1_	0.03877	0.05502	0.04844
	*Q_e_*	19.7897	33.7230	49.9256
	R^2^	0.9970	0.9959	0.9971
Quasi-secondary model	*k*_2_	0.00117	0.0012	0.00066
	*Q_e_*	27.4322	43.6677	66.1599
	R^2^	0.9978	0.9973	0.9993
Elovich model	*α*	2.6000	6.2009	8.1156
	*β*	0.2031	0.1160	0.0787
	R^2^	0.9472	0.9701	0.9654

**Table 2:** Adsorption isotherm model fitting parameters of UiO-66-NH_2_/SA@PEI.

Langmuir model	*Q_m_*	*K_L_*	R^2^
	96.7882	0.0275	0.9680
Freundlich model	*K_F_*	1/*n_F_*	R^2^
	9.3226	0.4431	0.9901
Temkin model	*K_T_*	*B*	R^2^
	0.4552	18.0698	0.9470
D–R model	*Q_m_*	*K_D_*	R^2^
	70.3320	2.3737×10^−5^	0.8157

**Table 3:** Thomas model for dynamic adsorption capacity of UiO-66-NH_2_/SA@PEI under different conditions.

*C*_0_	*v*	*m*	*t_e_*	R	Thomas Model		
					*K_T_*	*Q*	R^2^
50	1	2	291	29.19	0.2531	1.4594	0.9951
50	2	2	182	26.54	0.3830	1.3494	0.9873
100	2	2	89	23.79	0.4622	1.1422	0.9896

**Table 4:** The phosphate adsorption capacities with different porous materials.

Materials	Specific Surface Area (m^2^/g)	Pore Size (nm)	Adsorption Capacity (mg/g)	Ref.
UiO-66	990	——	27.70	[39]
UiO-66-NH_2_	815	——	30.00	
MFC@UiO-66	——	——	7.83	[40]
Mg-doped UiO-66-NH_2_	397	1.96	68.00	[41]
Ce-doping UiO-66-NH_2_	557.7	1.96	69.10	[42]
UiO-66-NH_2_/SA@PEI	325.1	3.7	68.75	This work

## Data Availability

Data is contained within the article.
